# MTA3 Regulates Extravillous Trophoblast Invasion Through NuRD Complex

**DOI:** 10.3934/medsci.2017.1.17

**Published:** 2017-01-16

**Authors:** Ying Chen, Sok Kean Khoo, Richard Leach, Kai Wang

**Affiliations:** 1 Department of Biomedical Sciences, Grand Valley State University, Allendale, MI, USA; 2 Department of Cell and Molecular Biology, Grand Valley State University, Grand Rapids, MI, USA; 3 Department of Obstetrics, Gynecology and Reproductive Biology, Michigan State University, Grand Rapids, MI, USA; 4 Department of Animal Science, Michigan State University, East Lansing, MI, USA

**Keywords:** MTA3, epigenetics, placenta, trophoblast, invasion

## Abstract

Extravillous trophoblast (EVT) invasion is required for remodeling uterine tertiary arteries and placenta development during pregnancy. Compromised EVT invasion may contribute to the pathology of placenta-related diseases. Metastasis -associated protein 3 (MTA3) is one of the subunits of nucleosome remodeling and deacetylation (NuRD) complex that represses transcription in a histone deacetylase-dependent manner. MTA3 is reported to be down-regulated in preeclamptic placentas, suggesting its potential role in EVT invasion. Here, we investigate the role of MTA3 in EVT invasion by studying its molecular mechanisms in EVT cells. First, we confirmed MTA3 expression in the EVT cells in human placenta using immunohistochemistry. We then used lentivirus-mediated MTA3 short hairpin RNA (shRNA) to knock down MTA3 expression in EVT-derived HTR8/SVneo cells and found higher invasion capacity in MTA3 knockdown cells. Using quantitative real-time PCR, we showed higher expression of invasion-related genes matrix metalloproteinase 2 (*MMP2*), matrix metalloproteinase 9 (*MMP9*), and transcription factor *Snail* in MTA3 knockdown compared with control cells. Co-immunoprecipitation-Western blot assay showed the protein-protein interaction of histone deacetylase 1 (HDAC1), a subunit of NuRD, with MTA3 in HTR8/SVneo cells. Co-immunoprecipitation-Mass spectrometry assay further identified 71 proteins interacting with MTA3, including NuRD subunits, heterochromatin proteins, epigenetics modifiers and transcription factors. This result not only indicated the involvement of NuRD complex in MTA3’s function, but also demonstrated the complicated multiple co-players in MTA3 and NuRD complex mediated transcription repression in EVT. In summary, our data demonstrates that MTA3 regulates EVT invasion and related gene expression via NuRD complex in EVT.

## 1. Introduction

Extravillous trophoblast (EVT) invasion is required for remodeling uterine tertiary arteries and placenta development [1,2]. Compromised EVT invasion is known to be related with the pathology of placenta-centered diseases such as preeclampsia and intrauterine growth restriction (IUGR) [3–5]. Invasion-related genes such as transcription factor Snail [6], matrix metalloproteinase 2 (*MMP2*) [7] and matrix metalloproteinase 9 (*MMP9*) [8] have been reported to play critical roles in EVT invasion and dysregulations of these genes may contribute to the etiology of preeclampsia and IUGR [9–11].

Epigenetics is an important gene regulation mechanism in placenta development and trophoblasts differentiation [12]. As part of epigenetic mechanism, chromatin remodeling is essential for trophoblast lineage differentiation and placenta function. Metastasis associated protein 3 (MTA3), a subunit in a chromatin remodeling complex called nucleosome remodeling and deacetylation (NuRD) [13], is expressed in placental EVT [14]. MTA3 is deregulated in preeclamptic placentas [15], suggesting its potential role in EVT invasion.

In our previous study, we showed MTA3 regulates trophoblast gene expression in villous trophoblast [15]. A recent report identified MTA3 functions in stem cells [16]; MTA3 regulates the differentiation of cytotrophoblast stem cells into EVT cells [16]. However, the role of MTA3 and its related mechanisms in EVT, especially EVT invasion, is still largely unknown.

Here, we investigate the role of MTA3 in EVT invasion and its related gene expression. Additionally, we use a proteomics approach to identify protein-protein interaction of MTA3 during transcription repression process.

## 2. Materials and Methods

### 2.1. Immunohistochemistry (IHC) analysis of MTA3 in placental EVT

Four millimeter-thick placenta sections from eight placentas were obtained from the Human Female Reproductive Tract Biorepository at Michigan State University (MSU) Center for Women’s Health Research. Slides were dewaxed in xylene, rehydrated in a gradient ethanol series and subjected to antigen unmasking with a PH 9.0 buffer (Vector Laboratories, Burlingame, CA). Samples were incubated with primary antibody against MTA3 (Abcam, Cambridge, MA; Table 1) overnight at 4°C, followed by incubation with biotin-conjugated secondary antibodies, and then horseradish peroxidase conjugated Streptavidin (Vector). MTA3 protein signals were detected after exposure to diaminobenzidine (DAB). This study was approved by MSU institutional review board.

### 2.2. Cell culture and lentivirus mediated knockdown and overexpression

HTR8/SVneo cell line was a gift from Charles H. Graham [17] and was authenticated by positive cytokeratin expression, invasion capacity and related gene expression (data not shown). HTR8/SVneo cells were cultured in DMEM/F12 supplemented with 10% FBS, 2 mmol/L L-glutamine and 1% penicillin and streptomycin. Lentivirus coding shRNA against MTA3 mRNA was purchased from Applied Biosystems (Foster City, CA; Clone ID: V3LHS_360589, 360590, 360591; shRNA sequence: 5′-GCATTAAAGCAGCGTATC-3′, 5′-GCATTAAAGCAGCGTATC-3′ and 5′-GCATTAAAGCAGCGTAT-3′) and used in MTA3 knockdown experiments. Lentivirus coding MTA3 mRNA with a V5 tag (MTA3V5) was used for MTA3 overexpression in HTR8/SVneo cells in the co-immunoprecipitation assay according to our previously published protocols [18]. HTR8/SVneo cells were transfected by adding 3 μl lentivirus into each well of cultured trophoblasts and passaged for a minimum of 5 times in the presence of 5 ng/ml puromycin (approximately 2 weeks). HTR8/SVneo cells transfected with Lentivirus against a non-specific target were used as controls.

### 2.3. Invasion assay

2×10^4^ HTR8/SVneo cells were seeded in the upper layer of transwell membrane with Matrigel-coating (BD Biosciences, Franklin Lakes, NJ) for invasion assay. DMEM/F12 with 0.1% BSA and DMEM/F12 with 20% FBS were loaded in the upper and lower chamber respectively. Cells were fixed and stained with 0.09% crystal violet solution after 21 hours. Non-invading cells on the upper layer of the transwell membrane were removed by cotton swab. Images were taken from invading cells on the lower layer of the membrane and total number of cells was counted in three independent fields in a blinded manner. 3 biological replicates were conducted in this assay.

### 2.4. mRNA expression by quantitative real time PCR (RT-PCR)

Total RNA was isolated using RNeasy mini kit (Qiagen, Valencia, CA) according to the manufacturer’s protocol. Trace level DNA was eliminated with RNase-free DNase I (Qiagen). Total RNA was then reverse-transcribed into cDNA with Superscript II First-Strand Synthesis System (Invitrogen, Foster City, CA). Power SYBR Green PCR reagent (Applied Biosystems) was used for RT-PCR and gene expression was analyzed using delta Ct (cycle threshold) analysis. We investigated the gene expression of invasion-related genes matrix metalloproteinase 2 (*MMP2*), matrix metalloproteinase 9 (*MMP9*), and transcription factor *Snail* (Primers for each target gene are listed in Table 2). 3 biological replicates were conducted in this assay.

### 2.5. Protein expression by Western blot

Whole protein from HTR8/SVneo cells were isolated using RIPA buffer with protease inhibitor cocktail (Invitrogen) and quantified using bichinchoninic acid (Thermo Fisher Scientific, Foster City, CA). Ten micrograms of protein were loaded in each well of 10% reducing gel before transferred to PVDF membrane. Antibody information of MTA3 and Actin were listed in Table 1.

### 2.6. Co-immunoprecipitation (co-IP) Western blot and mass spectrometry

HTR8/SVneo cells overexpressing MTA3 with V5 tag (MTA3V5) were harvested and whole protein was extracted by adding NETN buffer with protease inhibitor cocktail (Invitrogen) before protein quantification using bichinchoninic acid (Thermo Fisher Scientific). Isolated protein was flash-frozen in liquid nitrogen until future use. For co-IP Western blot assay, 1 mg of whole protein and 2 μg of MTA3 rabbit antibody or non-specific rabbit IgG were used for each IP reaction (Table 1). IgA coated magnetic Dynabeads (Invitrogen) were used to pull down MTA3 and its associated proteins. The pull-down proteins were collected in lysis buffer after boiling for 5 mins and detected by Western blot procedure. For co-IP mass spectrometry, 25 mg of whole protein from 2 biological samples and 10 μg MTA3 antibody or non-specific IgG were used for IP reaction. MTA3 associated proteins were pulled down using IgA coated magnetic beads and digested by trypsin at 37°C for 6 hours. Peptide supernatant was removed and concentrated by solid phase extraction using OMIX tips (Agilent Technologies, Santa Clara, CA) according to the manufacturer’s protocols. Digested peptides were injected into an EASY-nLC 1000 liquid chromatograph (Thermo Fisher Scientific) by an Thermo Acclaim PepMap 100 0.1 mm × 20 mm C18 trapping column (Thermo Fisher Scientific) and washed for 5 min. Bound peptides were then eluted from the trapping column into an Acclaim PepMap RSLC 0.075 mm × 250 mm C18 analytical column (Thermo Fisher Scientific) for the separation process. Separated peptides were sprayed into a Q-Exactive mass spectrometer (Thermo Fisher Scientific) using a FlexSpray spray ion source. Survey scans were taken in the Orbitrap (70,000 resolution at m/z 200) and top ten ions in each survey scan were then subjected to automatic higher energy collision induced dissociation (HCD) with fragment spectra acquired at 17,500 resolution. The resulting MS/MS spectra were converted to peak lists using Mascot Distiller v2.5.1 (Matrix Science, Boston, MA), searched against a custom database containing human protein sequences (www.uniprot.org) and appended using the Mascot searching algorithm v 2.5. The Mascot output was then analyzed using Scaffold v4.5.0 (Proteome Software, Portland, OR) to probabilistically validate protein identifications. Protein assignments validated with Scaffold at 1% FDR confidence filter are considered to be true.

### 2.7. Statistical analysis

Student’s t-test was used to statistically analyze the invasion and quantitative RT-PCR data. A *p*-value of less than 0.05 was considered statistically significant.

## 3. Result

### 3.1. MTA3 is expressed in placental EVT and represses EVT invasion

**Figure 1:**
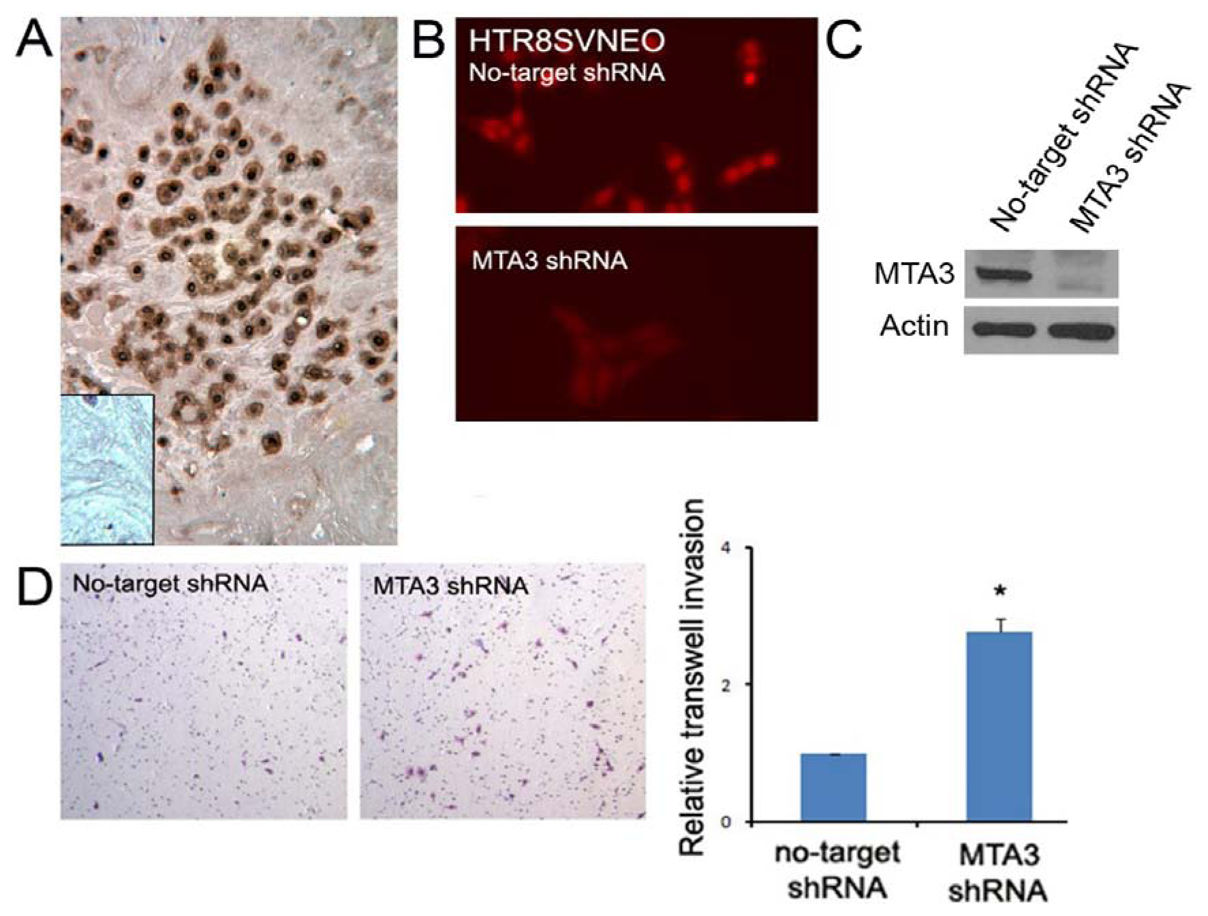
MTA3 repressed EVT invasion (A) MTA3 expression in EVT in the decidua layer of human full term placenta by IHC staining (MTA3 stained brown). Insert is negative control. (B, C) Knockdown of MTA3 in HTR8/SVneo cells shown in the Western blot and IF. (D) Knockdown of MTA3 increased HTR8/SVneo cell invasion by matrigel barriered transwell invasion assay.

Using IHC, MTA3 expression was detected in the EVT cells in the human full term placenta (Figure 1A). In the well-established trophoblast cell line HTR8/SVneo [17], MTA3 expression was knocked down by transfection with lentivirus coding for shRNA against MTA3 mRNA and verified by immunofluorescence (IF) (Figure 1B) and Western blot (Figure 1C). In Matrigel invasion assay, inhibition of MTA3 in MTA3 knockdown cells increased EVT invasion (2.8 fold) compared with control cells (Figure 1D).

### 3.2. MTA3 regulates invasion-related genes in EVT

**Figure 2:**
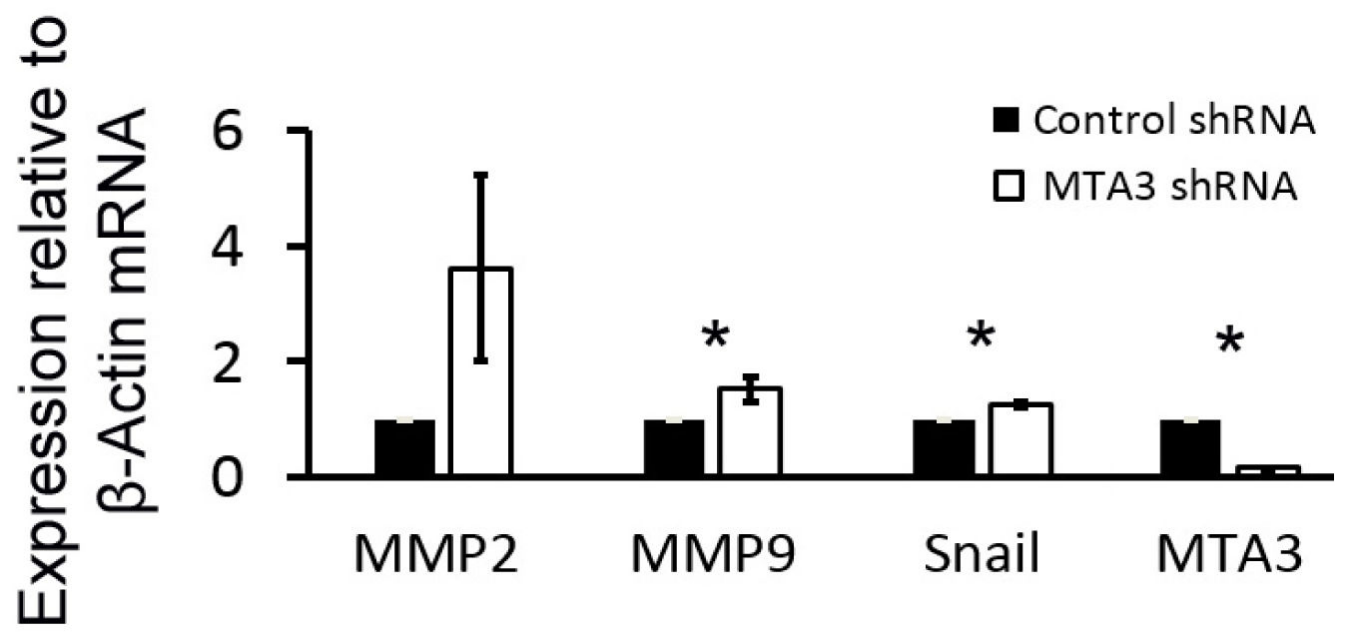
MTA3 regulated invasion genes MMP2, MMP9, Snail and MTA3 mRNA levels in MTA3 knockdown cells were compared with control cells using quantitative RT-PCR. These gene expressions were normalized with beta-actin mRNA level. Asterisk represented the statistical significance (*p* < 0.05) compared with the control.

To investigate the molecular mechanisms of MTA3 in EVT invasion, gene expression of invasion-related genes *MMP2*, *MMP9* and transcription factor *Snail,* as well as *MTA3,* were evaluated in MTA3 knockdown cells using quantitative RT-PCR. We showed statistically significant down-regulation of *MTA3* (> 84%) and up-regulation of *MMP9* and *Snail* in MTA3 knock-down cells compared with control cells (Figure 2). We also detected increased MMP2 expression, though it did not reach significance.

### 3.3. MTA3 is associated with NuRD complex in EVT

**Figure 3:**
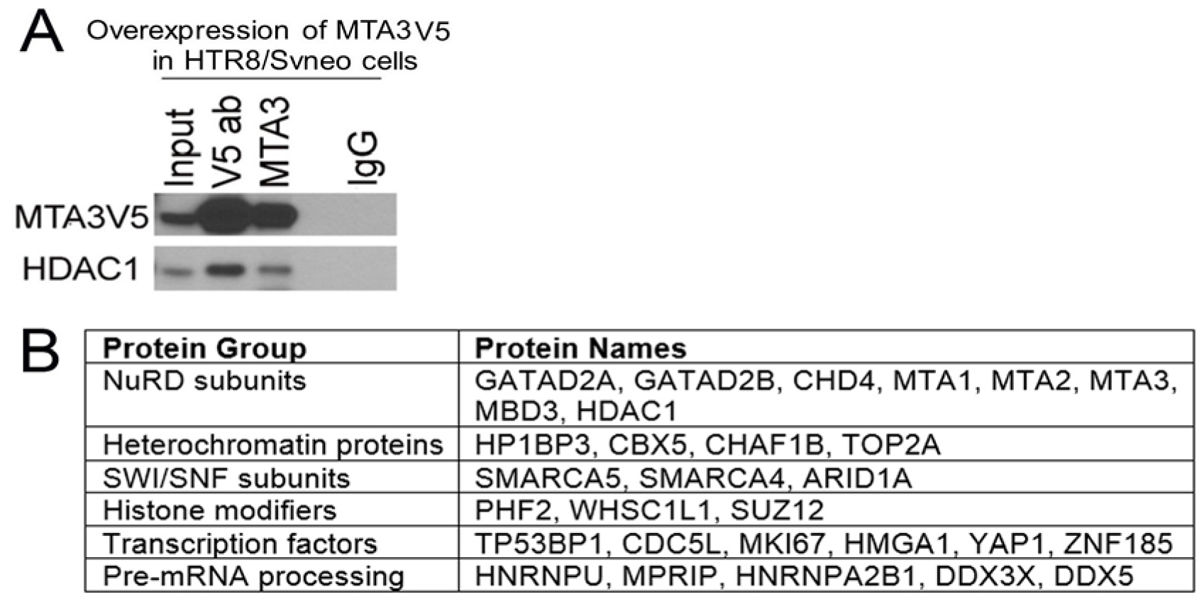
MTA3 associated with NuRD subunits in EVT (A) MTA3 was associated with HDAC1, a NuRD subunit, in HTR8/SVneo cells demonstrated by IP-Western blot. MTA3 with V5 tag (MTA3V5) was overexpressed in HTR8/SVneo cells. (B) Representative MTA3 associated proteins by co-IP-mass spectrometry.

To further understand the mechanisms of MTA3 transcription regulation, we identified the protein-protein interactions of MTA3 in transcription repression process. MTA3 with V5 tag (MTA3V5) was over-expressed in HTR8/SVneo cells. Co-IP Western blot and mass spectrometry were used to identify MTA3 associated proteins in these cells. In co-IP-Western blots, MTA3 was pulled down and enriched by both MTA3 and V5 antibodies when compared with IgG control antibody (Figure 3A). HDAC1, a subunit of NuRD complex, was identified in MTA3 and V5 pull-downs, indicating the association of MTA3 with NuRD complex in EVT cells. Co-IP-mass spectrometry further identified 71 MTA3-associated proteins in MTA3 pull-down cells (Table S1). MTA3-associated proteins include NuRD subunits (e.g. MTA1, MTA2, MTA3, MBD3 and CHD4), heterochromatin proteins (e.g. CBX5), transcription factors (e.g. YAP1), SWI/SNF proteins (e.g. SMARCA5), and pre-mRNA processing proteins. Representative protein groups were listed in Figure 3B.

## 4. Discussion

Dynamic reconstruction of epigenome happens in the trophoblast differentiation and function and chromatin remodeling proteins such as MTA3 are known to play important roles in this process [19]. MTA3 is a subunit of NuRD complex and is involved in the regulation of villous trophoblast differentiation and fusion [15,16]. In this study, we showed inhibition of *MTA3* correlates with increased trophoblast invasion and up-regulated invasion-related genes *MMP2*, *MMP9*, and *Snail* in EVT cells. In addition, 71 interacting proteins for MTA3 were identified. These proteins include NuRD subunits, heterochromatin proteins, epigenetics modifiers, and transcription factors.

EVT cells play an important role in embryo implantation and maternal-fetal homeostasis and EVT invasion is critical to pregnancy success. Here, we used HTR8/SVneo, a cell line derived from first trimester cytotrophoblasts immortalized by overexpression of SV40 antigen [17], to study the effect of MTA3, a transcriptional co-repressor known to regulate gene expression in placental trophoblasts [15], in EVT invasion. In the EVT invasion process, EVT cells are required to degrade the extracellular matrix of the basal layer. Matrigel, a gelatinous protein mixture secreted by mouse sarcoma cells which is broadly used to mimic the extracellular matrix, is used in our *in vitro* invasion assay. Knockdown of MTA3 in EVT-derived cells accelerates cell invasion, indicating the role of MTA3 in EVT invasion. *MMP2* and *MMP9* are two major matrix metalloproteinases and involve in digesting extracellular matrix in placenta; loss of function of *MMP2* and *MMP9* are associated with un-remodeling spiral vessel, hypertension, compromised trophoblast invasion [20] and preeclampsia during pregnancy [21]. Transcription factor *Snail* is known to regulate *MMP*s [7,22] and up-regulated MMPs and Snail are associated with increased trophoblast invasion [7,23]. In this study, we found knockdown of *MTA3* significantly increased *MMP9* and *Snail* gene expression. Thus, higher invasion capability of EVT cells within *MTA3* knockdown EVT derived cells can be explained by the up-regulation of *MMP9* and *Snail*.

To further understand the mechanism of MTA3 mediated transcription repression in EVT invasion process, we used co-IP-mass spectrometry to investigate the protein-protein interaction of MTA3. We identified 71 MTA3-associated proteins that include NuRD subunits, heterochromatin proteins, epigenetics modifiers, transcription factors, and pre-mRNA processing proteins. These results showed a unique and specific NuRD subunit set interacting with MTA3 in EVT cells. Enrichment in heterochromatin proteins agreed with the major function of MTA3 which is transcription repression. SWI/SNF chromatin remodeling proteins in MTA3 interacting protein list suggested the collaborative function between NuRD and SWI/SNF complexes in certain genes [24]. Having epigenetic modifiers as MTA3 interacting proteins proposed collaborative effort of different epigenetic mechanisms in the transcription repression process of EVT cells. We identified 40 transcription factors that are associated MTA3, suggesting complex combinatorial control of MTA3 transcription in the NuRD complex of EVT cells.

In summary, our results suggest that MTA3 regulates EVT invasion through NuRD complex. Understanding the molecular mechanisms of MTA3 in EVT cells may provide deeper insights about the etiology of preeclamptic placentation during pregnancy.

## Supplementary Material

Table S1

## Figures and Tables

**Table 1:** Antibody information.

Antibody target	Company/Source	Catalog	Application and Dilution
MTA3	Abcam	AB87275	WB 1:1000; IP: 0.4 ug/mg IHC: 1:200
V5 antibody	Invitrogen	R96025	IP: 0.4ug/mg WB 1:1000
Control IgG	Bethyl	P120-101	IP: 0.4ug/mg IHC: 1:200
HDAC1	Cell Signaling	5646	WB: 1:2000
Actin	Sigma	A5441	WB 1:10000

**Table 2:** Primer sequences.

Gene symbol	NCBI GI/ID	F/R	Primer sequence (5′ to 3′)	Position to TSS or in cDNA
MMP2	GI:700274109	F	GAAGAAGAAAATGGATCCTGG	1951
		R	GAAGAAGTAGCTGTGACCGCCGC	2065
MMP9	GI:74272286	F	GGGCTACGTGACCTATGACA	2101
		R	GTATCCGGCAAACTGGCTCC	2222
Snail	GI:301336132	F	GGCGTGTGCTCGGACCTTCT	801
		R	AGGCAGGGGCAGGTATGGAG	920
MTA3	GI:50878291	F	CCTAATAAGAAGGATAGAAGAACTC	281
		R	CTGCGAGCATTATAAGTGTGTTG	393
Actin	GI:168480144	F	CAGCAGATGTGGATCAGCAAG	1141
		R	TTGTCAAGAAAGGGTGTAACGC	1252
